# Diabetes health care specific services readiness and availability in Kenya: Implications for Universal Health Coverage

**DOI:** 10.1371/journal.pgph.0002292

**Published:** 2023-09-27

**Authors:** Stephen N. Onteri, James Kariuki, David Mathu, Antony M. Wangui, Lucy Magige, Joseph Mutai, Vyolah Chuchu, Sarah Karanja, Ismail Ahmed, Sharon Mokua, Priscah Otambo, Zipporah Bukania

**Affiliations:** Centre for Public Health Research, Kenya Medical Research Institute Kenya, Nairobi, Kenya; University of Minnesota, UNITED STATES

## Abstract

Diabetes is a major cause of morbidity and mortality worldwide yet preventable. Complications of undetected and untreated diabetes result in serious human suffering and disability. It negatively impacts on individual’s social economic status threatening economic prosperity. There is a scarcity of data on health system diabetes service readiness and availability in Kenya which necessitated an investigation into the specific availability and readiness of diabetes services. A cross sectional descriptive study was carried out using the Kenya service availability and readiness mapping tool in 598 randomly selected public health facilities in 12 purposively selected counties. Ethical standards outlined in the 1964 Declaration of Helsinki and its later amendments were upheld throughout the study. Health facilities were classified into primary and secondary level facilities prior to statistical analysis using IBM SPSS version 25. Exploratory data analysis techniques were employed to uncover the distribution structure of continuous study variables. For categorical variables, descriptive statistics in terms of proportions, frequency distributions and percentages were used. Of the 598 facilities visited, 83.3% were classified as primary while 16.6% as secondary. A variation in specific diabetes service availability and readiness was depicted in the 12 counties and between primary and secondary level facilities. Human resource for health reported a low mean availability (46%; 95% CI 44%-48%) with any NCDs specialist and nutritionist the least carder available. Basic equipment and diagnostic capacity reported a fairly high mean readiness (73%; 95% CI 71%-75%) and (64%; 95%CI 60%-68%) respectively. Generally, primary health facilities had low diabetic specific service availability and readiness compared to secondary facilities: capacity to cope with diabetes increased as the level of care ascended to higher levels. Significant gaps were identified in overall availability and readiness in both primary and secondary levels facilities particularly in terms of human resource for health specifically nutrition and laboratory profession.

## Introduction

The burden of non-communicable diseases (NCDs) among people aged 30–70 years has increased progressively especially in developing countries [1, 2], exerting additional pressure on an already constrained health system by the demands of infectious diseases [3]. As of 2020, about 41 million annual deaths accounting for 74% of all global deaths were attributed to NCDs of which 2 million resulted from diabetes or its complications [4].

Diabetes, like most NCDs, results from a combination of genetic, physiological, behavioral and environmental factors [3, 5]. Other identified disease drivers include aging population, rapid urbanization and globalization of unhealthy lifestyle [6]. Diabetes is among the leading cause of renal failure, limb amputation, blindness and a major trigger of cardiovascular disease which is a leading cause of death in diabetic patients [7]. The complications of undetected, untreated and unmanaged diabetes result in serious human suffering and disability. Furthermore, they negatively impact on individual’s social economic status resulting from huge financial implications that extend beyond health, threatening economic prosperity: trapping people into poverty, denying them a dignified life, undermining their ability to work and be productive [8].

In Kenya, NCDs are estimated to account for 27% (284,000) of all deaths, with diabetes being responsible of about 10,000 deaths [9, 10]. In 2021, International Diabetes Federation reported Kenyan diabetes related mortality at approximately 15, 284 persons among individuals aged between 20–79 years [11, 12]. In 2013, the adjusted prevalence of diabetes in Kenyan adults was estimated at 3.6% which is projected to rise to 4.4% if no mitigations efforts are enforced by 2035 [13].

One major challenge in prevention, treatment and management of diabetes in Kenya according to STEPwise Survey of 2015 is undiagnosed cases. The report indicated that more than 88% of Kenyans have never had their blood sugar tested, resulting to late diagnosis hence high morbidity and mortality in addition to increased cost of disease management [14]. Achieving universal access to NCD services requires the active engagement of all the stakeholders to prevent new cases by increasing population and service coverage right from primary health facilities to referral levels [15]. Attainment of Universal Health Coverage (UHC) for NCD services will require increased investment [16]; for preventive care including development of a minimum package of targeted NCD services and integrating this within Ministry of Health (MoH) routine programmes [15]; additionally, successful delivery of preventive and curative care for NCDs requires multi-sectoral programs, partnerships and collaborations [16, 17].

Now is the time to deliver on Sustainable Development Goals (SDGs), especially goal 3 target 3.4: to reduce by one third premature mortality from non-communicable diseases through prevention and treatment and promote mental health and well-being by 2030 [18]. This achievement will contribute to the attainment of SDG 1. Even though developing countries, including Kenya, have put up efforts in this regard, governments still need to invest more on health system readiness and availability to improve prevention, control, diagnosis and management of NCDs in addition, to the implementation of NCDs WHO “best buys” and other action plans [19].

To address the growing burden of NCDs, more systematic and methodically robust approaches are required. Critical to this is investing in better management of NCDs, which includes early detection, screening, treating and provision of access to different forms of care such as palliative care for those in need. Strengthening primary health care approach can be a highly impactful essential NCD intervention bolstering early detection and timely treatment. Existing evidence suggests that timely provisions of such interventions to patients are excellent economic investments in the reduction of future expensive treatments [4].

In Kenya, limited studies have been conducted to assess the service readiness and availability of healthcare facilities in terms of diabetes management capacity. A comprehensive assessment of healthcare facilities’ diabetes specific service availability and readiness is thus critical to identify existing challenges and gaps which will inform strategies for strengthening of the health system. Against this backdrop, a study was conducted by the Kenya Medical Research Institute aimed at investigating health systems capacity and readiness, populations’ perceptions, needs, health service satisfaction and their impact on Universal Health Coverage in 12 counties in Kenya. A sub-analysis from the study was done to assess the health facilities’ service availability and readiness to provide diabetes specific services in the 12 counties.

## Methodology

### Ethics statement

Ethical standards outlined in the 1964 Declaration of Helsinki and its later amendments were upheld throughout the study. The research was conducted with integrity and transparency ensuring the welfare and rights including confidentiality, privacy, autonomy and cultural diversity of all participants and stakeholders involved in the study were upheld.

### Health system setting in Kenya

Health system in Kenya operates under the stewardship of the Ministry of Health (MoH). The Kenya Essential Package of Health (KEPH) defines services and interventions to be delivered and the tiers or levels of health system. Service delivery is organized in six KEPH levels: Level 1: Community health services, Level 2: Dispensaries, Level 3: Health Centers, Level 4: Sub-County Hospitals, Level 5: County Referral Hospitals and Level 6: National Referral Hospitals [20]. The selection of study facilities took into considerations KEPH level classification. Level 2, 3 and 4 were considered and also facility ownership in this case public facilities-owned by and managed by the Ministry of Health, Municipal Authorities and government institutions) [20].

### Study design

A convergent parallel mixed method study design was used to investigating health systems capacity and readiness, populations’ perceptions, needs, health service satisfaction and their impact on Universal Health Coverage in 12 counties in Kenya. For diabetes specific readiness and availability, a sub-analysis of the quantitative data from the main study was utilized.

### Study site

**Fig 1:**
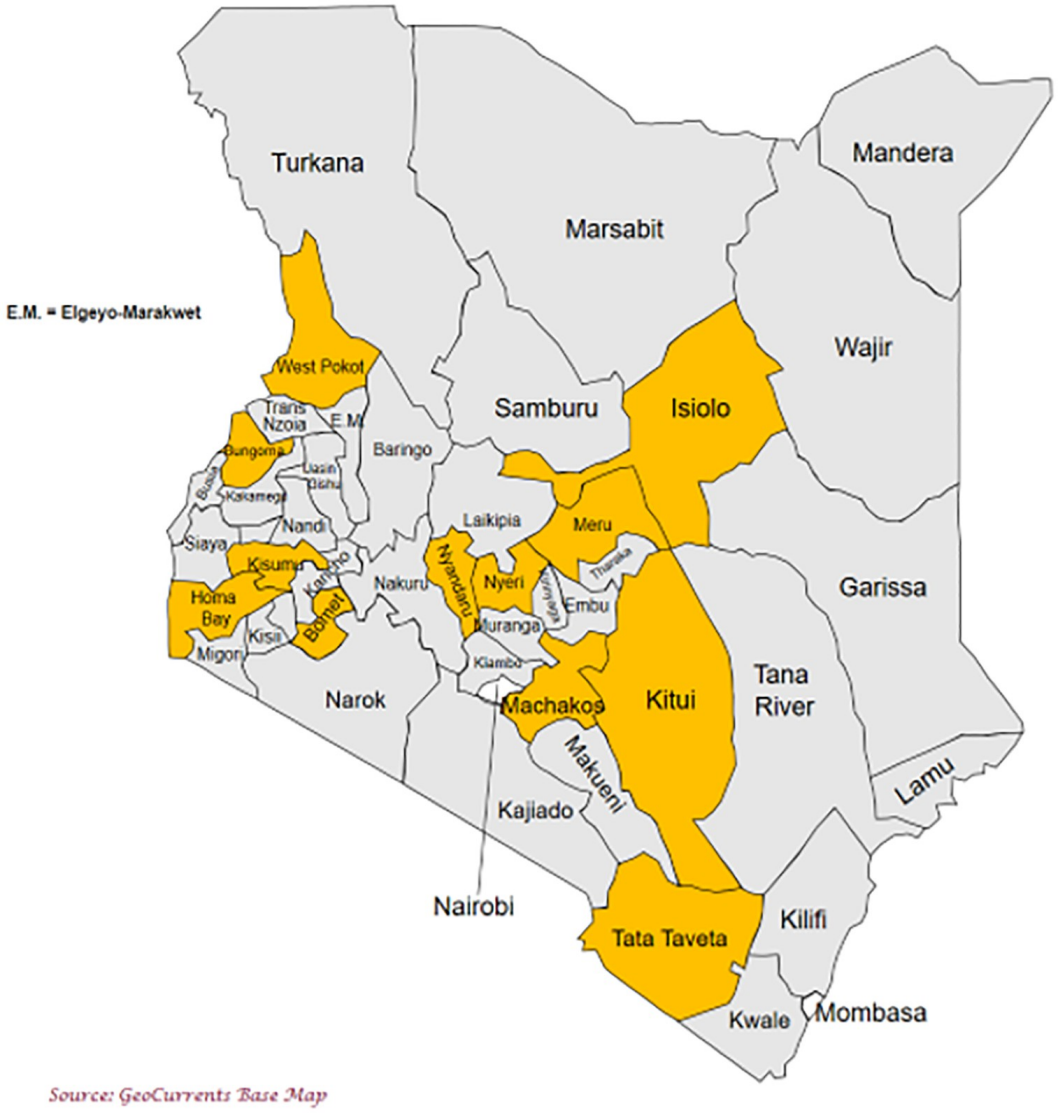
Study counties on the Kenyan map. Source link: KISM, Nairobi, Kenya, 2021.

The study was conducted in 12 of the 47 counties in Kenya: Isiolo, Kisumu, Nyeri, Machakos, Bomet, Bungoma, Homabay, Kitui, Meru, Nyandarua, Taita Taveta and West Pokot in Kenya (Fig 1).

### Data source, data collection and participants

Data was collected between February 2020-March 2020 and October 2020-December 2020 from randomly selected health facilities from all tiers as described in the health system setting section above. The Kenya service availability and readiness mapping (SARAM) tool designed to assess service availability and readiness of the health sector was administered [20] to either the facilities heads/managers or in-charges (preferred) or a senior member of staff with sufficient knowledge of the facility capacity and operations. In the event that any of the members stated above could not be reached, the study team approached the next person in the hierarchy.

The data collection process was automated using Open Data Kit (ODK) platform. The SARAM tool was uploaded into the platform and administered. The questionnaire aimed to assess the readiness and availability of health facilities in various aspects, including human resources for health, physical infrastructure resources, availability of basic equipment, basic amenities and assets, essential medicines and medical supplies, diagnostic capacities and the provision of basic healthcare interventions including non-communicable conditions.

### Variables

Health care service availability and readiness scores are the standard WHO indicator of health system capacity to provide health care services. To deduce readiness and service availability for diabetes, specific SARAM domains were analyzed. For service availability, which is the physical presence [21] human resource for health that is the presence of nutrition professional, laboratory professionals, nursing professionals and the availability of any NCDs specialist were evaluated by calculating the percentage proportion of facilities (primary or secondary) that reported presence. Availability of any NCD specialist evaluation was excluded in primary facilities as they are not part of the cadres expected at that level.

For service readiness which refers to the health system capacity to provide or deliver service by having critical health components [21], basic facility equipment (height meter, weight meter and Bp machine), basic laboratory equipment (glucometer machine and glucometer strips), range of service available (which included diagnosis or management of NCDs such as diabetes), laboratory diagnosis (Blood glucose, urine dipstick glucose and urine protein test), health promotion including health education on nutrition, prevention of NCDs and drug and substance abuse were analyzed.

### Sample size

Sample size was calculated by estimating the minimum sample size required to estimate a proportion with a pre-defined desired level of precision putting into consideration adjustments to take care of non-response, design effect and correction for finite populations [22]. A sample size of 791 health facilities was obtained which included both public and private facilities. For diabetes specific service availability and readiness, only public facilities were considered.

### Sampling

Twelve counties of Isiolo, Kisumu, Nyeri, Machakos, Bomet, Bungoma, Homabay, Kitui, Meru, Nyandarua, Taita Taveta and West Pokot were randomly sampled. Four UHC pilot counties Isiolo, Kisumu, Machakos and Nyeri were each paired with two counties purposively selected one being neighboring which was likely to have similar morbidity patterns and high possibility of being affected by the UHC piloting and the other far off which was unlikely to have patient’s crossover to pilot counties for treatment (Table 1).

**Table 1:** Study counties included in the study.

	Pilot County	Neighboring County	Distant Counties
1	Kisumu	Homa Bay	Bungoma
2	Machakos	Kitui	Taita Taveta
3	Nyeri	Nyandarua	West Pokot
4	Isiolo	Meru	Bomet

At the county level, health facilities were stratified by ownership and KEPH levels. Square root allocation method [23] was used in each respective county to determine the number of public health facility required. Simple random sampling method with a random start was then used to select the facilities involved per strata.

### Data management

An Open Data Kit (ODK) source tool which is a digital data gathering platform was used and inbuilt data quality checks were employed. Data was uploaded into the cloud server and password protected. Data was backed up regularly to avoid any loss or tampering and all study documentation and data files were access controlled for confidentiality. The principal investigator was the sole custodians of all data (hard and soft copies).

### Data analysis

Data cleaning and validation was done prior to statistical analysis using IBM SPSS version 25. Assessed level 2, 3 and 4 facilities were categorized into primary level facilities and this included (level 2 and 3) first point of contact and secondary level facilities (level 4) responsible for curative and rehabilitative services at analysis level. To uncover the distribution structure of continuous study variables as well as identify outliers or unusually entered values, exploratory data analysis (EDA) technique was employed. For continuous variables, descriptive statistics was used. For categorical variables descriptive statistics in terms of proportions, frequency distributions and percentages were used.

### Data quality assurance

The data collection tools were pretested in a pilot study. The database design had in-built data quality checks. These included “must answer” prompters and skip patterns which were built-into the database. During data collection, the data manager routinely carried out data validation by profiling sample data for completeness and consistency. Any error identified was reported to the team lead for follow up and correction or verification.

Apart from administering a structured questionnaire, the data team also observed physical presence of the assessed items that were observable at the time of study. The data collectors recruited were bachelor degree holders with at least one year experience in scientific research data collection. Before, data collection, the research assistants were trained on data collection instrument and data entry using the Open ODK tool.

### Ethical considerations and entry plan

Scientific and ethical approval was granted by the Kenya Medical Research Institute’s Scientific and Ethical Review Unit (SERU) (KEMRI/SERU/CPHR/OO5/3945). In addition, research permit was obtained from the National Council of Science, Technology & Innovation (NASCOTI).

The study sought a letter of support from the State Department of Health, Ministry of Health (MOH) to conduct the study in the 12 counties. Local approval to conduct the study was obtained from the health County Executive of Committee (CEC) of the respective counties. Upon receipt of approval, entry to the selected health facilities and the community was facilitated by the County Director of Health services and Community Health Strategy Focal Persons (CHFP) who sensitized and mobilized the health facility in charges through the Sub-County Health Management Teams (SCHMT).

### Participant consent

Written informed consent to participate in the study was sought from all eligible participants in a language well understood to them, prior to their participation in the study. Participant’s confidentiality and privacy were respected.

## Results

Out of 648 eligible public health facilities, a total of 598 were visited in 12 counties as indicated in Table 2 representing a response rate of 92%. The proportion of level 2, 3 and 4 facilities were 57.4%, 25.9%, and 16.6% respectively. The facilities not visited were either not operational or were inaccessible during the study period.

**Table 2:** Public facilities visited & assessed.

County	Public Facilities Visited & Assessed by KEPH Level			
	Primary Care		Secondary Care	Total
	Level 2	Level 3	Level 4	
Bungoma	22	10	10	42
Homa Bay	26	19	15	60
Isiolo	16	4	2	22
Kisumu	15	8	23	46
Kitui	38	15	13	66
Machakos	34	15	4	53
Meru	26	12	13	51
Nyandarua	28	21	2	51
Nyeri	33	18	4	55
Taita Taveta	24	12	5	41
West Pokot	42	8	4	54
Bomet	40	13	4	57
**Overall Total**	**344**	**155**	**99**	**598**

### Selected basic equipment for diabetes readiness

One of the main functions of health system is provision of access to quality health service that responds to client their demand. Facilities in different tiers should have service readiness in various aspects of the health system. In regards to selected basic equipment for diabetes readiness, height and weight meters, blood pressure (BP) machine, glucometer machine and glucometer strip were analysed at primary and secondary facilities. Height and weight meters are critical for measuring the Body Mass Index (BMI) a key diabetes indicator derived from the patient’s weight in kilograms (kg) and height in meters (m). Increased BMI is normally associated with the risk of type 2 diabetes, and other NCDs [21]. Studies have shown elevated blood pressure value is common among people with diabetes. In fact, more than two-thirds of diabetic patient have been found with elevated blood pressure hence the need to assess readiness in facilities regarding blood pressure (Bp) measures [24]. Furthermore, the development of hypertension has also been reported to coincide with high blood sugar levels [25]. The glucometer machine plus glucometer strip are important in measurement of blood sugar levels informing risk of disease occurrence or severity.

Overall, glucometer machine plus strips was reported least in most facilities at primary level with only 45% of facilities reporting readiness. For secondary facilities, weighing scale digital was the least reported with 82% of facilities reporting readiness.

At the primary level, Homa Bay and Taita Taveta counties reported the lowest and highest overall average readiness of specified diabetes tracer equipment at 51% and 82% respectively. In Meru County, only 53% of examined primary institutions reported preparedness with height and weight meters shown in Table 3.

**Table 3:** Basic diabetic equipment readiness in primary and secondary facilities.

County	Category	N	Weighing scale (digital) (%)	Bp Machine digital (%)	Height and weight meter (%)	Glucometer With strips (%)	Ave. Readiness Proportion (%)
**Bungoma**	Primary	32	59.4	75.0	75.0	59.4	67.2
	Secondary	10	100.0	100.0	90.0	90.0	95.0
**Homa Bay**	Primary	23	60.0	68.9	64.4	11.1	51.1
	Secondary	23	66.7	93.3	86.7	80.0	81.7
**Isiolo**	Primary	53	70.0	95.0	75.0	65.0	76.3
	Secondary	13	50.0	100.0	100.0	100.0	87.5
**Kisumu**	Primary	49	56.5	87.0	87.0	39.1	67.4
	Secondary	4	60.9	100.0	87.0	82.6	82.6
**Kitui**	Primary	38	77.4	92.5	83.0	39.6	73.1
	Secondary	13	100.0	100.0	92.3	92.3	96.2
**Machakos**	Primary	49	69.4	95.9	77.6	38.8	70.4
	Secondary	2	75.0	100.0	100.0	100.0	93.8
**Meru**	Primary	51	71.1	94.7	52.6	60.5	69.7
	Secondary	4	92.3	92.3	84.6	100.0	92.3
**Nyandarua**	Primary	36	63.3	93.9	83.7	65.3	76.5
	Secondary	5	100.0	100.0	100.0	100.0	100.0
**Nyeri**	Primary	50	72.5	94.1	78.4	58.8	76.0
	Secondary	4	100.0	100.0	100.0	100.0	100.0
**Taita Taveta**	Primary	53	86.1	88.9	86.1	66.7	81.9
	Secondary	4	100.0	100.0	100.0	100.0	100.0
**West Pokot**	Primary	45	86.0	50.0	80.0	24.0	60.0
	Secondary	15	75.0	100.0	100.0	100.0	93.8
**Bomet**	Primary	20	62.3	84.9	75.5	35.8	64.6
	Secondary	2	100.0	100.0	100.0	100.0	100.0
**TOTAL**	Primary	499	70.1	84.6	76.6	45.3	69.1
	Secondary	99	81.8	98.0	90.9	90.9	90.4

Blood glucometer machine plus glucometer strip were generally the least reported in all counties, with Homa Bay and West Pokot County reporting the lowest proportion of facilities in primary level possessing the items at 11% and 24%, respectively.

In general, most secondary facilities (level 4) across the 12 counties reported high readiness in regards to the assessed tracer equipment (height meter, weight meter, Bp machine, glucometer machine, and glucometer strips). Only, Homa Bay, Kisumu and Isiolo Counties reported a readiness score below 90% that is 82%, 83% and 88% respectively (Table 3).

### Laboratory tests diagnostic readiness

Rapid diagnostic testing using blood glucose, urine dipstick glucose, and urine protein test were studied for diabetes laboratory diagnosis. This is inconsideration of the fact that blood glucose and urine glucose levels are indicators of insulin functionality, which can be measured in the blood or urine directly. Extra glucose (blood glucose levels above normal ranges) in the blood is frequently eliminated through the urine, making a urine glucose test an indicator of a high glucose level [26]. Protein urine testing is generally used to detect kidney impairment caused by an excess of protein in the urine. Diabetes is characterized by kidney dysfunction, which is directly linked to insulin resistance [27, 28].

**Fig 2:**
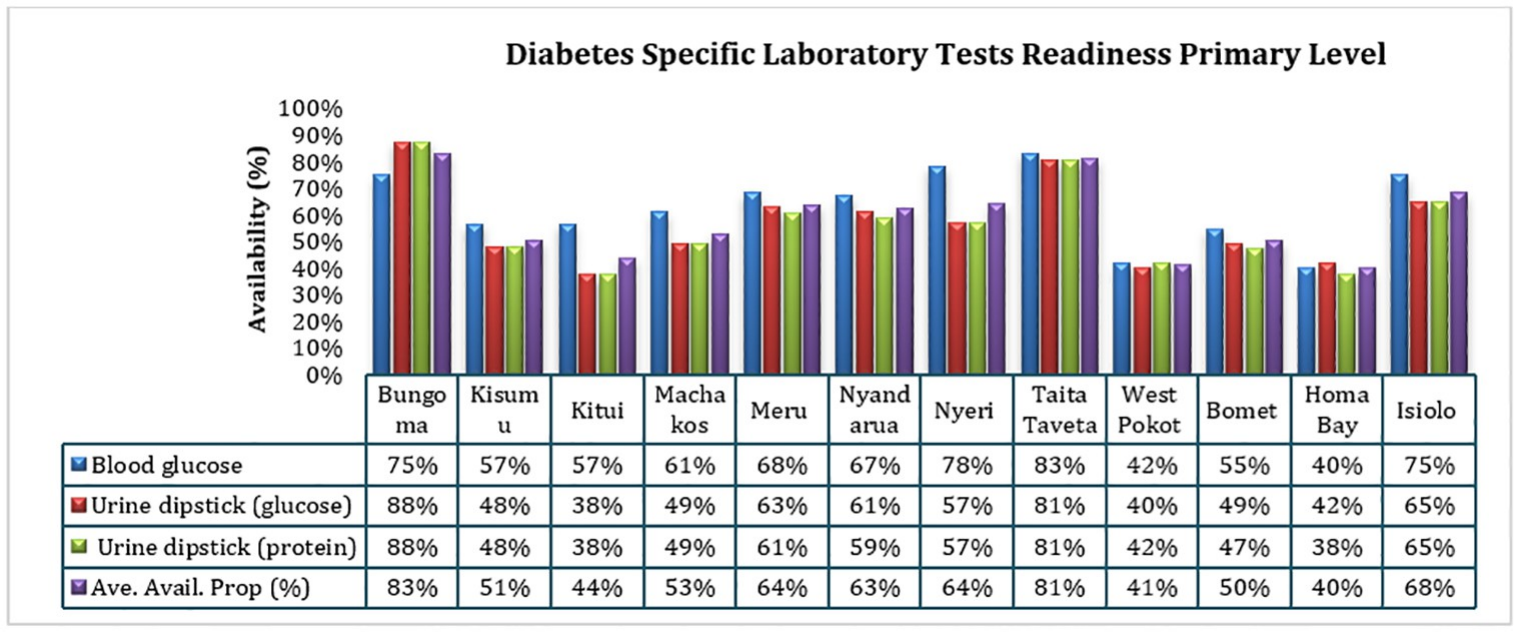
Basic laboratory tests readiness in primary care.

Secondary health facilities reported a readiness of over 90% across the study counties for each of the laboratory test service. For primary facilities, out of the 12 study counties, only Kitui County (44%), West Pokot County (41%) and Homa Bay County (40%) reported readiness average below 50%. Bungoma County reported the highest readiness average at 83%. Overall, urine dipstick protein and glucose reported least readiness in the study area as shown in Fig 2.

### NCDs diagnosis and management service readiness

Diagnosis or management of NCDs including diabetes was assessed in the study. The study respondent reported on the presence or absence (yes or no) of the service.

**Fig 3:**
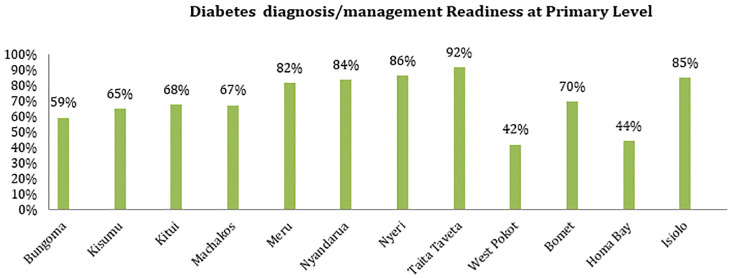
Diabetes diagnosis/management readiness at primary level.

All secondary facilities assessed in the study area reported readiness in diagnosis and management of diabetes. For primary level facilities, majority of the counties reported service readiness in more than half of the assessed facilities, except for West Pokot and Homa Bay Counties where only 42% and 44% of their assessed facilities reported readiness respectively Fig 3.

### Health promotion and education readiness

Health promotion and education is vital in provision of knowledge and awareness regarding disease prevention and management. It enables the population to have more control over health determinants taking meaningful action to reduce risk factors, and support health and wellbeing for all [29]. It is embedded in the Kenya health system and Ministry of Health has developed guidelines and standards which outline the minimum standards at which health promotion practice and training should be conducted [30]. In the study, health promotion of NCDs prevention, nutrition education and drug and substance abused were considered. The presence or absence of these services was assessed.

Majority of the health reported fair readiness in health promotion and education in most of the domains. It is important to note that no secondary level facilities in Isiolo reported availability of health promotion and education in regards to drug and substance abuse contrarily to 75% of primary facilities which reported readiness. Similarly, primary facilities were more ready to provide NCDs prevention health promotion and education (95%) compared to secondary level (50%) in the county (Table 4).

**Table 4:** Health promotion and education service readiness.

County	KEPH Level	N	Nutrition Education (%)	Prevention of NCDs Health Education (%)	Drug and Substance Abuse (%)	Ave. Readiness Proportion (%)
**Bungoma**	Primary	32	90.6	93.8	90.6	91.7%
	Secondary	10	100.0	90.0	100.0	96.7
**Kisumu**	Primary	23	78.3	78.3	78.3	78.3
	Secondary	23	87.0	87.0	69.6	81.2
**Kitui**	Primary	53	83.0	81.1	81.1	81.7
	Secondary	13	100.0	100.0	100.0	100.0
**Machakos**	Primary	49	87.8	89.8	75.5	84.4
	Secondary	4	100.0	100.0	75.0	91.7
**Meru**	Primary	38	89.5	92.1	84.2	88.6
	Secondary	13	92.3	92.3	84.6	89.7
**Nyandarua**	Primary	49	89.8	91.8	85.7	89.1
	Secondary	2	100.0	100.0	100.0	100.0
**Nyeri**	Primary	51	88.2	90.2	86.3	88.2
	Secondary	4	100.0	100.0	75.0	91.7
**Taita Taveta**	Primary	36	94.4	91.7	86.1	90.7
	Secondary	5	100.0	80.0	60.0	80.0
**West Pokot**	Primary	50	86.0	90.0	78.0	84.7
	Secondary	4	100.0	100.0	75.0	91.7
**Bomet**	Primary	53	83.0	88.7	79.2	83.6
	Secondary	4	100.0	100.0	75.0	91.7
**Homa Bay**	Primary	45	100.0	97.8	95.6	97.8
	Secondary	15	100.0	100.0	93.3	97.8
**Isiolo**	Primary	20	90.0	95.0	75.0	86.7
	Secondary	2	100.0	50.0	0.0	50.0

### Specific human resource for health on permanent employment and were present (available) at the time of the assessment-availability

One of the health sectors pillars is human resource for health. In the study, availability of a clinical officer, nursing profession, pharmacists, nutritionist, laboratory profession, and any NCDs specialist were analysed. Availability of NCDs specialist at primary level was not assessed as they are not expected to be at the level.

**Fig 4:**
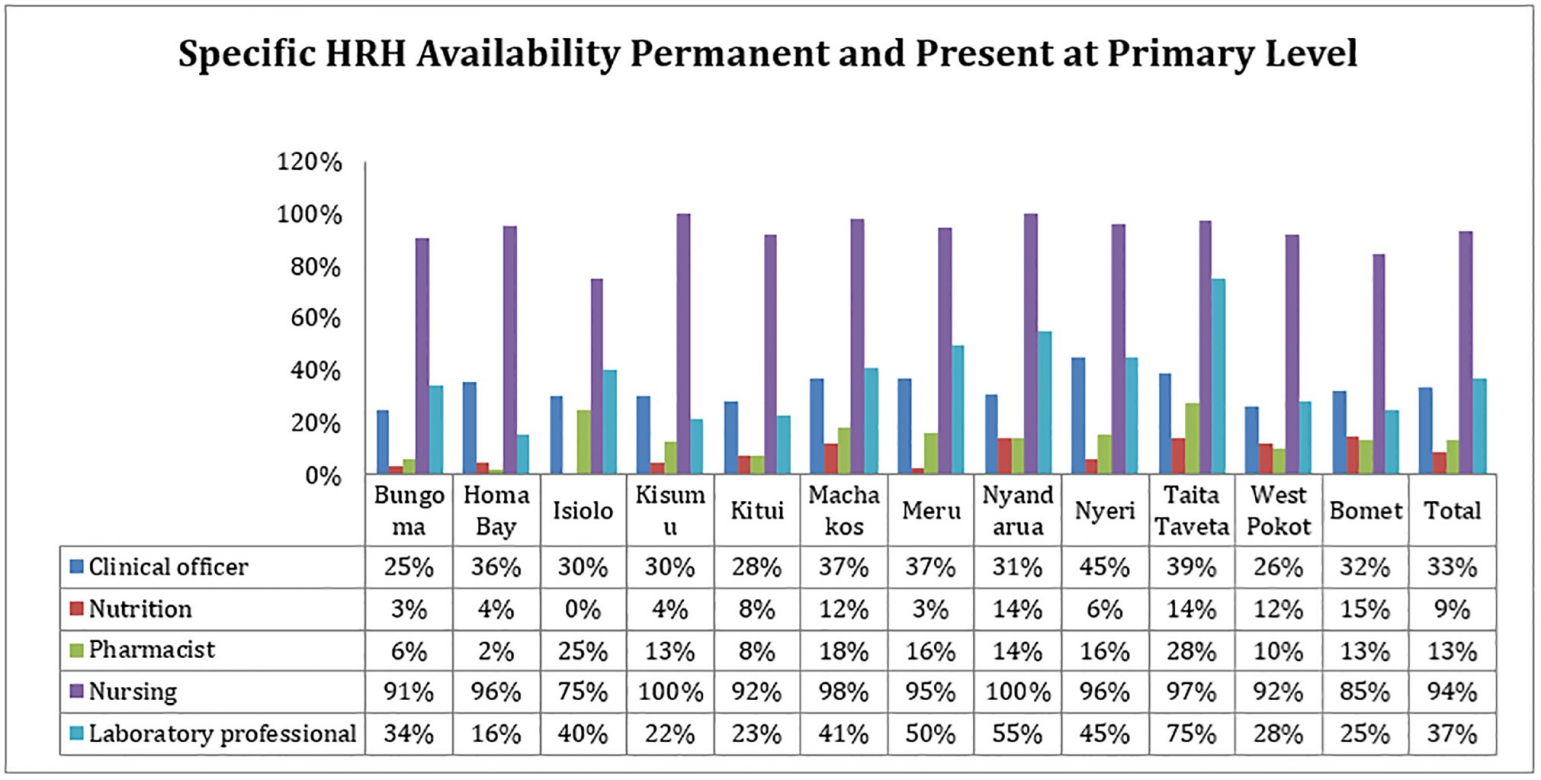
Specific HRH availability permanent and present at primary level.

Nursing profession was highly reported in primary level facilities. For nutrition profession, more than 80% of facilities across the counties reported unavailability. Bomet County reported the highest number of facilities with nutrition professionals with 15% of assessed facilities in the county reporting availability. For laboratory profession, only Taita Taveta (75%), Nyandarua (55%), and Meru (50%) counties reported availability of the profession in more than half of the facilities assessed. Taita Taveta county reported the highest proportion of facilities with pharmacist (28%) with the rest of the counties reporting poor availability as more than 75% of the facilities reported unavailability as shown in Fig 4.

**Fig 5:**
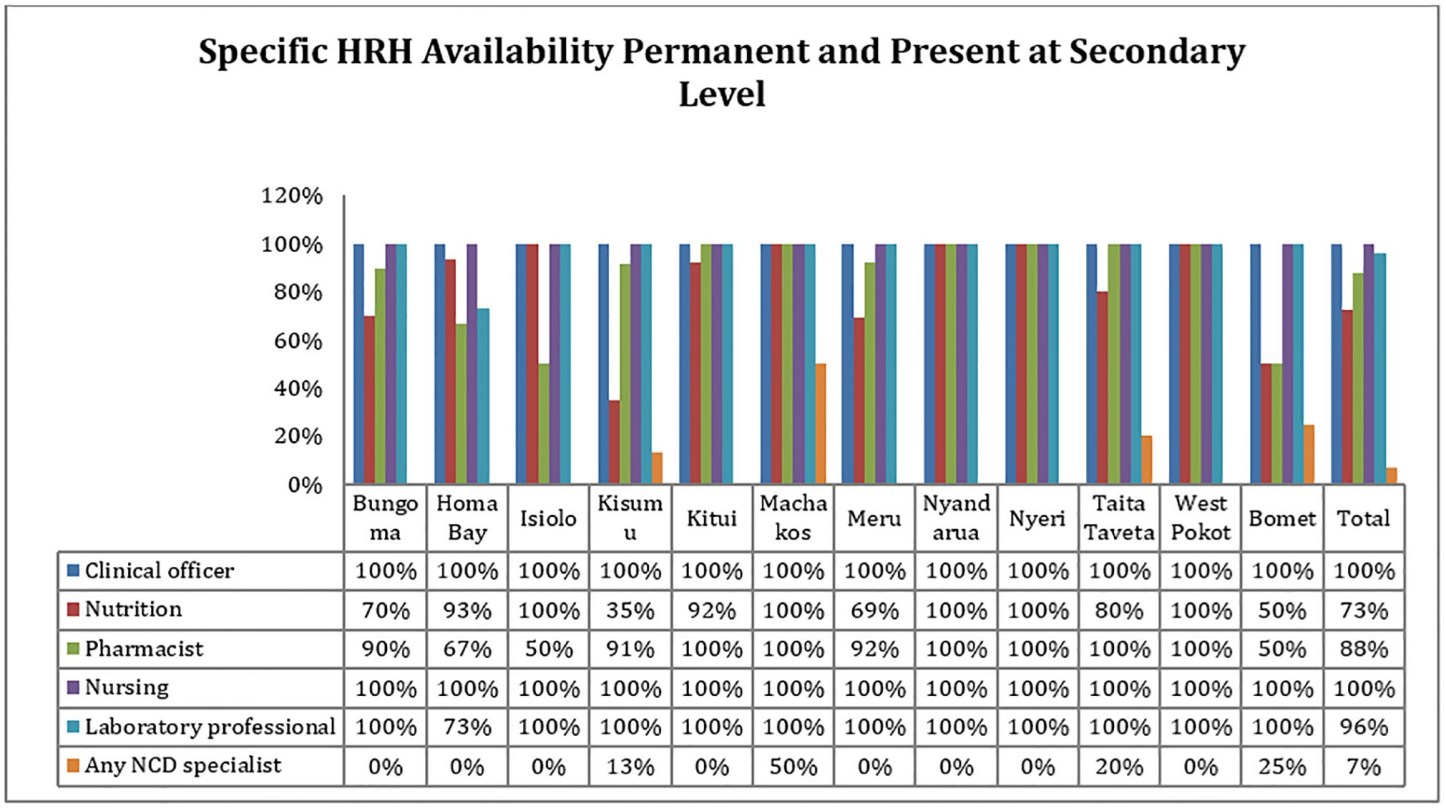
Availability of required HRH at secondary level who were present and employed on permanent basis.

In the secondary level, nursing profession and clinical officer were the most reported available across (100%) the counties. Laboratory profession was also highly reported in majority of the counties (100%) except in Homa Bay County where availability was reported at 73%. Availability of any NCD specialist was low across the 12 counties, except for Machakos County which reported availability in at least 50% of the assessed health facilities (Fig 5).

### Diabetes services availability and readiness index

The WHO’s SARA guide was used in analyzing specific diabetes readiness and availability [31, 32]. Readiness indicators for each item under main readiness domain were recorded as binary variables, taking value “1” for the availability of tracer item and “0” for the absence of items in the facility. Generally, there was poor diabetes specific services readiness and availability based on all the assessed service domains in the 12 counties with all the counties reporting a mean below 15%. For diabetes diagnosis/management Taita Taveta County reported the highest mean availability at 93% while West Pokot reported the least mean availability (46%). Despite a fair readiness in regards to basic equipment and diagnosis capacity, majority of county health facilities lacked sufficient human resources to utilize the available resource as shown in Table 5.

**Table 5:** Readiness and availability index and domain scores for diabetes by counties.

Services for diabetes	Bungoma (n = 42)	Homa Bay (n = 60)	Isiolo (n = 22)	Kisumu (n = 46)	Kitui (n = 66)	Machakos (n = 53)	Meru (n = 51)	Nyandarua (n = 51)	Nyeri (n = 55)	Taita Taveta (n = 41)	West Pokot (n = 54)	Bomet (n = 57)	Total (n = 598)
**SERVICE AVAILABILITY**													
**Diabetes diagnosis / management**	69.0	58.3	86.4	82.6	74.2	69.8	86.3	84.3	87.3	92.7	46.3	71.9	74.6
**SERVICE READINESS**													
**Staff and guidelines** *													
**Clinical officer**	42.9	51.7	36.4	65.2	42.4	41.5	52.9	33.3	49.1	46.3	31.5	36.8	44.3
**Nutrition**	19.0	26.7	9.1	19.6	24.2	18.9	19.6	17.6	12.7	22.0	18.5	17.5	19.4
**Pharmacist**	26.2	18.3	27.3	52.2	25.8	24.5	35.3	17.6	21.8	36.6	16.7	15.8	25.8
**Nursing**	92.9	96.7	77.3	100.0	93.9	98.1	96.1	100.0	96.4	97.6	92.6	86.0	94.6
**laboratoty technician**	50.0	30.0	45.5	60.9	37.9	45.3	62.7	56.9	49.1	78.0	33.3	29.8	47.0
**Staff domain index; mean (95% CI)**	46.2 (35.8–56.6)	44.7 (37.0–52.4)	39.1 (24.5–53.7)	59.6 (50.5–68.6)	44.8 (36.3–53.4)	45.7 (37.1–54.2)	53.3 (44.4–62.2)	45.1 (37.2–57.0)	45.8 (38.0–53.7)	56.1 (47.1–65.0)	38.5 (30.9–46.2)	37.2 (29.0–45.4)	46.2 (43.7–48.7)
**Equipment**													
**Weigh-scale digital**	69.0	61.7	68.2	58.7	81.8	69.8	76.5	64.7	74.5	87.8	85.2	64.9	72.1
**Bp machine**	81.0	75.0	95.5	93.5	93.9	96.2	94.1	94.1	94.5	90.2	53.7	86.0	86.8
**Height-weight meter**	78.6	70.0	77.3	87.0	84.8	79.2	60.8	84.3	80.0	87.8	81.5	77.2	78.9
**Glucometer with strips**	66.7	28.3	68.2	60.9	50.0	43.4	70.6	66.7	61.8	70.7	29.6	40.4	52.8
**Equipment domain index; mean (95% CI)**	73.8 (64.9–82.7)	58.8 (51.8–65.7)	77.3 (66.0–88.6)	75.0 (68.7–81.3)	77.7 (72.1–83.2)	72.2 (64.6–79.8)	75.5 (69.0–82.0)	77.5 (69.3–85.6)	77.7 (71.5–84.0)	84.1 (77.6–90.7)	62.5 (56.2–68.8)	67.1 (59.5–74.7)	72.7 (70.6–74.7)
**Diagnostics**													
**Blood glucose**	81.0	55.0	77.3	78.3	65.2	64.2	76.5	68.6	80.0	85.4	46.3	57.9	68.2
**Urine dipstick glucose**	90.5	56.7	68.2	71.7	50.0	52.8	72.5	62.7	60.0	82.9	44.4	52.6	62.0
**Urine dipstick protein**	88.1	53.3	68.2	73.9	50.0	52.8	70.6	60.8	60.0	82.9	46.3	50.9	61.4
**Diagnostics domain index; mean (95% CI)**	86.5 (77.0–96.0)	55.0 (43.5–66.5)	71.2 (51.7–90.7)	74.6 (62.8–86.5)	55.1 (43.6–66.5)	56.6 (43.5–69.7)	73.2 (61.0–85.4)	64.1 (50.9–77.2)	66.7 (55.2–78.2)	83.7 (72.2–95.3)	45.7 (33.0–58.3)	53.8 (41.3–66.3)	63.9 (60.3–67.4)
**Readiness index for diabetes; mean (95% CI)**	61.7 (53.8–69.7)	56.3 (48.6–64.0)	61.7 (49.1–74.3)	63.7 (56.0–71.4)	56.5 (49.9–63.1)	54.5 (46.7–62.2)	62.8 (55.8–69.7)	66.7 (59.4–74.1)	58.9 (52.3–65.6)	74.3 (66.9–81.7)	42.2 (35.6–48.8)	53.6 (45.6–61.5)	58.7 (56.5–60.9)
**% Of facilities with ALL services (availability & readiness services) for diabetes**	11.9	10.0	4.5	2.2	9.1	7.5	5.9	11.8	3.6	14.6	1.9	3.5	7.2

* Staff and guidelines; Only the staff in this domain were assessed.

## Discussion

Physical accessibility to quality services which integrates with clients’ demand is one of the main functions of a health system. Health care facilities should have the capacity to provide the services recommended at different tiers that is service readiness and service availability.

As expected, secondary level facilities had a higher readiness and availability compared to primary level facility in all the assessed domains. However, critical gaps were reported especially in human resource: availability any NCDs specialist. Suboptimal readiness and availability were reported in primary level posing a major challenge in early detection/diagnosis and management of diabetes as the facilities at the level are the first point of contact with patients offering preventive and curative care. The study findings depict a common trend especially in developing countries where health systems face significant challenges in readiness and availability to deal with NCDs including diabetes. The finding of this study concurs with a study conducted in Nepal that found a lack of preparedness for services related to NCDs, including diabetes, in a significant number of rural public facilities [33]. Similarly, a systematic study on the readiness of Sub-Saharan Africa’s health care system for diabetic management found wide gaps in terms of availability and readiness [34].

Based on the assessed indicators including basic equipment, diagnosis capacity, health promotion including education a low mean readiness score (59%; 95% CI: 57–61) was obtained. This was slightly lower compared to a study done in 258 facilities in Kenya and which examined facilities readiness based on the availability of indicators such as equipment, diagnostic capacity, medicines and commodities, by Rita Ammoun et al. (2022) which reported that 82.2% of the facilities offered diabetes services. The mean readiness scores for diabetes reported in the study was (71%; 95% CI: 67–74) [35].

### Basic equipment to screen for diabetes risk factors

Secondary level facilities reported a fair average readiness in basic equipment (90%) while primary facilities (70%) lagged slightly behind as indicated by the study findings. This represents an increase of readiness of diabetes specific tracer equipment compared to findings reported by the Kenya Harmonized Facility Assessment (KHFFA) 2018/2019 study that reported a 63 percent readiness [36]. Notable gaps were however reported that require intervention especially in regards to blood glucometer machine and strip at primary level facilities (45%) and weighing scale digital at secondary level (82%). These findings correspond to a Kenyan national survey finding conducted between 2019–2020 by the African Population and Health Research Center (APHRC) which reported significant gaps in basic equipment readiness in facilities [37]. A multicounty report also reported notable gaps in diabetes basic equipment readiness with none of the facilities reporting presence of all 12 tracer equipment assessed which included blood pressure and adult scales [38]. The findings are consistent with majority of findings reported in Low and Middle income countries where basic equipment is suboptimal in most of specific services as highlighted by Huda et (2021) in a cross-sectional nationally representative survey in Afghanistan, Bangladesh and Nepal where the three countries reported suboptimal readiness in basic equipment for diabetes management [38].

### Selected laboratory diabetes diagnostic tests, health facility’s capability in diagnosis and management of diabetes

Basic laboratory diagnosis and test equipment readiness has been shown to positively impact on diabetes management through early diagnosis and interventions [39]. Most of the primary level facilities faced a shortfall in laboratory diagnosis and management of diabetes specific tests. Facilities in Kitui, West Pokot and Homa Bay county on average reported the least readiness (<50%). This can be attributed to poor readiness in basic equipment reported at primary level especially glucometer and strip which only 45% of assessed facilities at the level reported readiness. The situation is different for secondary level, where all the facilities reported over 90% readiness of tracer tests items. This indicates high readiness for early screening at secondary level and the need for improvement at primary level.

### Prevention of diabetes through targeted health promotion and education

Most primary and secondary level public health facilities offered health promotion and education services mainly on nutrition and education on prevention of NCDs. Over 80% of primary and 91% of secondary facilities offer this critical service. The only exception was Isiolo County which had low proportions of facilities offering health promotion and education. Given that diabetes, and by extension, NCDs, is a growing public health concern, the observed trends should be sustained in order to promote widespread awareness about the risk factors at both population and primary care levels.

### Availability of selected human resource for health

Human resource for health is a major health sector pillar for service intervention and inputs. It is however one of the main challenges in low and middle income countries (LMICs). Based on the study findings, health system in Kenya at primary level reported disproportionate lack of human resources in majority of the assessed cadres (clinical officer, nutritionist, pharmacist and laboratory technologist). Secondary level facilities reported a higher availability compared to primary with main challenges being observed in availability of NCDs specialist and Nutrition professions. The study finding comparing to other LMICs reported a low mean readiness (46%; 95% CI 44%-49%) as opposed to for instance a cross-sectional survey conducted in 266 health facilities across Bangladesh using the WHO SARA standard tool which also reported a suboptimal availability of health professions in health facilities in the region with a mean readiness of 65% in district hospitals [40] which in this case can be equated to primary public health facilities. The study finding implies that the primary level facilities are not ready to offer diabetes screening and management. Although, the services are fairly available at secondary level, there critical gaps that need to be filled to enhance diabetes readiness. The observations are consistent with those found in Peru [41, 42]. This implies that there’s limited access to early screening resulting in missed early diagnosis and/or suspected diabetes conditions.

### Implications for policy

The findings of this study have important policy implications for diabetes management and by extension NCDs control in Kenya. The strategic focus for UHC aspiration is to strengthen primary-level facilities so as to enable them offer preventive and promotive health services. Generally, the study findings indicated that primary level facilities were less prepared to manage diabetes based on the diabetes service availability and readiness domains assessed. UHC priority interventions to foster universal access to NCD services include; (1) increasing the proportion of people receiving such services; (2) expanding the package of NCDs services that are provided; and (3) reducing the financial burden of accessing these services. Thus, to bridge the gap between population and health care needs for diabetes and by extension NCDs, health facilities should be equipped with the relevant tools, drugs, and human resources which should come as a “complete unified package” for NCDs control. In addition, public health promotion activities and education have the potential to awaken a critical mass of the population to the reality that chronic conditions are as important as acute diseases, that they are often life-long, and that they have a strong link to lifestyle choices. These behaviors tend to predispose one to NCDs risk and are deeply rooted in societal norms. Awareness creation campaigns should ideally emphasize on having a cultural change regarding how society perceives health related wellness and risky behaviors.

## Conclusion

Although the country has made significant progress in improving diabetes healthcare services readiness and availability, there are still gaps that need to be addressed to improve service delivery. A variation in specific service availability and readiness was depicted in the 12 counties and between primary and secondary-level facilities. Majority of the primary level facilities were not ready to offer diabetes screening and management. They had suboptimal readiness scores and limited availability of services to manage diabetes based on the specific service domain assessed. Although, services are fairly availability at secondary level, there were critical gaps that need to be filled so to enhance diabetes readiness. Health care facilities ideally should have the capacity to provide the services recommended at different tiers that is service readiness and service availability. This would translate to early detection and initiation of treatment which is a critical step in averting or delaying the onset of diabetes and related complications.

### Recommendation

1. More support/resources are needed to strengthen primary health facilities capacity to improve diabetes service readiness and availability. They are the first point of care and are easily accessible to majority of the population. It is expected that functional health facilities at this level will be able to provide a variety of services, including early detection of conditions, screening, and referral, in addition to the delivery of other basic health care services.
2. National and County Governments need to make investment in human resource capacity especially nutritionist and diabetes care specialist as an urgent need.
3. Health promotion and education is readily available but there is need to bring in professionalism by hiring more nutritional professionals especially in primary health facilities.

### Survey limitation

The study did not comprehensively assess service readiness and availability of all diabetes tracers which includes clinical processes: capacity to provide annual HbA1C testing, LDL cholesterol testing, urine albumin creatinine ratio (UACR), screening for nephropathy, regular eye examination among others. Level 1 (community health facility), level 5 (county referral hospital) and level 6 (National referral facilities) were not included in the study. In regards to health promotion including education, the study did not assess the type of services offered, who offered the services and level of training. Further studies are needed to explore on the county governments health systems capacity to provide sustainable diabetes diagnosis and quality of care including health outcomes.
